# Analysis on Effects of Comprehensive Nursing Care Applied in Interventional Therapy for Patients with Liver Cirrhosis and Liver Cancer

**Published:** 2019-03

**Authors:** Yuli GOU, Jing YI, Mei JIANG, Chunhong CAO

**Affiliations:** 1. Department of Hepatopancreatobiliary Surgery, The Second Hospital of Dalian Medical University, Dalian 116027, P.R.China; 2. Department of Radiology, The Second Hospital of Dalian Medical University, Dalian 116027, P.R.China

**Keywords:** Liver cirrhosis, Interventional therapy, Comprehensive nursing care, Complication

## Abstract

**Background:**

This study aimed to investigate the effects of comprehensive nursing intervention in interventional therapy for patients with liver cirrhosis and liver cancer.

**Methods:**

Overall, 512 liver cirrhosis patients complicated with liver cancer receiving interventional therapy in the Department of Oncology of the Second Hospital of Dalian Medical University (Dalian, China) from March 2010 to March 2016 were retrospectively analyzed. Patients were divided into observation group (n=310) and control group (n=202). Comprehensive nursing intervention was applied to observation group and conventional nursing care was applied to control group.

**Results:**

The degrees of satisfaction before and after nursing intervention, quality-of-life scores, incidences of postoperative complications and survival rates at 20 months after operation of the two groups were compared. The degree of great satisfaction in observation group was significantly higher than that in control group (*P*<0.001). The quality-of-life scores of the patients in observation group were obviously higher than those in control group (*P*<0.001). The incidence of postoperative complications in observation group was significantly lower than that in control group (*P*<0.001). The survival rates in observation group was significantly higher than that in control group (*P*=0.035).

**Conclusion:**

The application of comprehensive nursing intervention in the interventional therapy for liver cirrhosis and liver cancer can notably improve the life quality of the patients, lower the incidence of postoperative complications and increase the survival rate, which is worthy of clinical popularization and application.

## Introduction

Liver cancer is one of the malignant tumors with the highest incidence rates in the world, of which the incidence rate is low in developed countries and relatively higher in developing countries. Meanwhile, its mortality rate is only next to those of gastric cancer and esophageal cancer (1). The disease may occur in people of all ages, with the main age of onset of 40–49 years old (2). There are more men with liver cancer than women, accounting for nearly 6/7.

The incidence rate of liver cancer is very high in China, accounting for about 42% around the globe, and it is increasing year by year, with approximately 600,000 new cases every year. Moreover, nearly 200,000 people die from liver cancer every year (3, 4).

The integrated treatment of traditional Chinese and western medicine, including surgery, chemoradiotherapy and aftercare with traditional Chinese medicine, is often used to treat patients with liver cancer. However, since the early clinical features of the patients with liver cancer are not significant, most of the patients are in the intermediate and advanced stage when their diagnosis is confirmed and have missed the best time for surgical treatments (5, 6). According to statistics, less than 30% of the liver cancer patients have operative indications (7). In addition, the cure rate and survival rate of the patients with advanced liver cancer are decreased greatly (8). Interventional therapy for liver cancer can significantly extend the survival time of the patients, which is an important means of treating liver cancer patients in the intermediate and advanced stage (9). However, the disadvantages of the therapy, such as tumor thrombus and blood vessel blockage, still cannot be ignored because they may lead to declined treatment effect or even ineffective treatment in the patients (10). Effective nursing care can improve the safety and efficacy of the interventional therapy (11).

Comprehensive nursing care, with nursing procedures as its core, is a brand-new nursing mode integrating the advantages of group nursing and primary nursing, as well as a perfect combination of various nursing measures such as nursing philosophy, nursing plan and nursing quality evaluation (12). It can conduct nursing intervention item by item according to the specific disease conditions of every patient and combine effective therapeutic methods with high-quality nursing modes, finally achieving the expected goals of treatment in patients with severe diseases (13). Moreover, the traditionally disease-centered nursing mode cannot satisfy the demands of increasing number of tumor patients (14).

Therefore, it was hypothesized in this experiment that the people-oriented comprehensive nursing intervention could improve the nursing quality of the patients, so its role in the effect of interventional therapy for patients with liver cirrhosis and liver cancer was investigated, and our viewpoints were proved through experiments.

## Materials and Methods

### Research objects

The case data of 512 liver cirrhosis patients complicated with liver cancer receiving interventional therapy in the Department of Oncology of The Second Hospital of Dalian Medical University (Dalian, China) from March 2010 to March 2016 were retrospectively analyzed. There were 438 males and 74 females aged 37–65 yr old, with an average age of (51.73±7.44) years old. All the patients were divided into observation group (n=310, comprehensive nursing intervention) and control group (n=202, conventional nursing care) according to the different nursing methods they received.

All the enrolled patients were diagnosed with scirrhous hepatocellular carcinoma through pathological sections in The Second Hospital of Dalian Medical University, had no past history of tumors, received a series of examinations and treatments in The Second Hospital of Dalian Medical University after the diagnosis was confirmed, were willing to cooperate with the arrangement of the health-care workers and had complete case history. Exclusion criteria: 1) patients complicated with other cardiovascular and cerebrovascular diseases, respiratory diseases or digestive tract diseases, 2) patients who transferred to other hospitals midway, 3) patients who took antibiotics prescribed by hospitals other than The Second Hospital of Dalian Medical University during the treatment or 4) patients who were treated with rehabilitation therapy arranged by other hospitals.

This research was approved by the Ethics Committee of The Second Hospital of Dalian Medical University, and informed consent for case collection was obtained from all the patients.

### Methods

Comprehensive nursing intervention was applied to observation group, and conventional nursing care was applied to control group. The nursing procedures in observation group were conducted by the nurses in strict accordance with the *2013 Operation Guide for Comprehensive Nursing Care* (15). The degrees of satisfaction before and after nursing intervention, quality-of-life scores, incidences of postoperative complications and survival rates at 20 months after operation of the two groups of patients were recorded separately.

### Evaluation criteria

Both complications and awareness rate of precautions were presented as ratios, and centesimal system was used for both satisfaction survey and quality-of-life scoring. The degree of satisfaction was divided into very satisfied (90 points or above), satisfied (60–80 points) and not satisfied (60 points or below), and the overall degree of satisfaction (60 points or above) of the patients was calculated. The quality-of-life scoring mainly included scoring of physical function, cognitive function, functions of social activities, no unhealthy emotions (anxiety, etc.), no disease recurrence and no pains. Higher scores stood for better conditions. The comprehensive evaluation was given after the patients and their families understood the precise meaning of each item.

### Statistical methods

Statistical methods: Statistical Product and Service Solutions (SPSS) 22.0 software (Chicago, IL, USA) was used for statistical analysis. The enumeration data were presented as *x̄*±*s*, and the measurement data were expressed by ratio. Chi-square test was adopted for comparison of measurement data, and *t*-test for enumeration data. *P*<0.05 suggested that the difference was statistically significant.

## Results

### General data

There were no statistically significant differences in gender, age, tumor-node-metastasis (TNM) staging and other basic data between observation group and control group. As for intra-group comparisons, the ratio of men was obviously higher than that of women in both groups (*P*<0.001). Similarly, the ratios of smoking and drinking patients were higher than those of non-smoking and non-drinking patients in the two groups (*P*<0.001). Smoking is harmful to health and drinking needs to be in moderation. Although the ratio of patients at stage III–IV was higher than that of patients at stage I–II in both groups, the difference was not statistically significant. It indicated that people pay more and more attention to their health, and the disease is discovered earlier. In the two groups, the proportion of patients with urban household was much higher than that of patients with rural household (*P*<0.001), and the proportion of patients without exercise habits far exceeded that of patients with exercise habits (*P*<0.05) (Table 1).

**Table 1:** Comparisons of clinical data of the two groups of patients [n (%)]

***Variable***	***Observation group (n=310) ***	***Control group (n=202) ***	***Statistical value***	***P***
Gender [n (%)]			0.413	0.720
Male ^a^	262 (84.52)	176 (87.13)		
Female	48 (15.48)	26 (12.87)		
Age(yr)	51.32±6.14	52.14±8.25	1.287	0.199
TNM staging [n (%)]			0.003	0.958
Stage I–II	145 (46.77)	94 (46.53)		
Stage III–IV	165 (53.23)	108 (53.47)		
Smoking [n (%)]			0.272	0.602
Yes ^b^	217 (70.00)	137 (67.82)		
No	93 (30.00)	65 (32.18)		
Drinking [n (%)]			1.306	0.253
Yes ^c^	248 (80.00)	153 (75.74)		
No	62 (20.00)	49 (24.26)		
Place of residence [n (%)]			1.546	0.214
City ^d^	239 (77.10)	165 (81.68)		
Countryside	71 (22.90)	37 (18.32)		
Exercise habit [n (%)]			1.523	1.234
No ^e^	239 (77.10)	146 (72.28)		
Yes	71 (22.90)	56 (27.72)		

Note: In both groups, the proportion of male

a is higher than that of female (*P*<0.05); the ratio of smoking

b patients exceeds that of non-smoking patients (*P*<0.001); the proportion of drinking

c patients is higher than that of non-drinking patients (*P*<0.001); the ratio of patients with urban household

d exceeds that of patients with rural household (*P*<0.05), and the proportion of patients without exercise habits

e is higher than that of patients with exercise habits (*P*<0.05)

### Degree of satisfaction of the patients

The degree of satisfaction with the nursing methods of the two groups of patients was recorded and analyzed. It was shown that the degree of satisfaction with comprehensive nursing care in observation group was not significantly different from that with conventional nursing care in control group. However, the degree of great satisfaction in observation group was 82.58% (n=256), which was remarkably higher than that in control group [33.17% (n=67), *P*<0.01]. It was seen that comprehensive nursing intervention can increase the patient’s degree of satisfaction notably (Table 2).

**Table 2:** Results of satisfaction survey [n (%)]

***Variable***	***Observation group (n=310) ***	***Control group (n=202) ***	***Statistical value***	***P***
Degree of satisfaction (%)	99.35	89.11	0.698	0.403
Very satisfied	256 (82.58)	67 (33.17)	31.75	<0.001
Satisfied	52 (16.77)	113 (55.94)	42.27	<0.001
Not satisfied	2 (0.65)	22 (10.89)	25.69	<0.001

### Quality-of-life score of the patients

The results of quality-of-life scoring of the two groups of patients indicated that the average score in observation group was (84.83±7.41) points, higher than that in control group [(64.30±9.14) points] (*P*<0.001). Moreover, the score of every item of quality-of-life scoring (including physical function, cognitive function and function of social activities) in observation group was superior to that in control group (*P*<0.001) (Table 3).

**Table 3:** Results of quality-of-life scoring (point)

***Variable***	***Observation group (n=310) ***	***Control group (n=202) ***	***Statistical value***	***P***
Average score	84.42±7.28	68.31±10.94	20.01	<0.001
Physical function	83.01±7.55	69.52±9.52	17.80	<0.001
Cognitive function	81.13±8.68	71.15±9.21	12.41	<0.001
Function of social activities	88.64±7.03	70.68±9.35	24.75	<0.001
No unhealthy emotion	81.98±8.54	60.78±10.97	24.49	<0.001
No disease recurrence	87.22±7.70	68.19±6.14	29.53	<0.001
No pains	84.56±8.16	69.52±8.98	19.59	<0.001

### Incidence of postoperative complications

The statistical results of the patients’ complication incidences after interventional therapy in the two groups showed that the incidence of postoperative complications in observation group was significantly lower than that in control group (21.6% *vs*. 48.5%, *P*<0.001).

### Survival rate at 20 months after operation

**Fig. 1:**
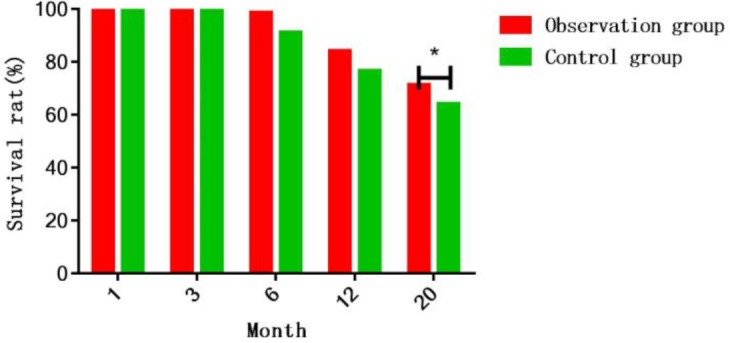
Survival rate at 20 months after operation of the two groups of patients It is indicated in the statistical results of the patients’ survival rates at 20 months after interventional therapy in the two groups that there are no statistically significant differences in the 1-, 3-, 6- and 12-month survival rates in the two groups of patients, while the survival rate at 20 months after operation in observation group is obviously lower than that in control group (71.3% *vs*. 64.2%, *P*<0.05)

It was indicated in the statistical results of the patients’ survival rates at 20 months after interventional therapy in the two groups that there were no statistically significant differences in the 1-, 3-, 6- and 12-month survival rates in the two groups of patients, while the survival rate at 20 months after operation in observation group was obviously lower than that in control group (71.3% *vs*. 62.4%, *P*=0.035) (Fig. 1).

## Discussion

Emphasizing on the people-oriented idea, with the patients and their disease demands as the directions, the comprehensive nursing intervention conducts comprehensive assessment of the patients’ physical fitness, mental status and disease severity and formulates targeted, organized and detailed nursing protocols, aiming to effectively improve the patients’ prognosis and life quality and decrease the occurrence of infections and complications (16, 17).

This experiment aims to study the application value of the comprehensive nursing intervention and provide reference and guidance for treatment of patients with liver cancer in future clinical practices by retrospectively reviewing the nursing efficacies of comprehensive nursing intervention and conventional nursing care in patients with liver cirrhosis and liver cancer, strictly requiring the nurses to perform the procedures and rigorously screening the research objects according to the inclusion and exclusion criteria.

The results of this experiment showed that both groups of patients were relatively satisfied with their nursing modes, and there was no difference in the degree of satisfaction between the two groups. However, the degree of great satisfaction with comprehensive nursing care of the patients in observation group was significantly higher than that with conventional nursing care in control group, indicating that the efficacy of comprehensive nursing care is still superior to that of conventional nursing care.

A study (18) revealed that among the currently existing nursing modes, the comprehensive nursing intervention is a set of scientific nursing mode with the characteristics of more apparent effects and higher degree of satisfaction in patients. In addition, it can improve the nursing quality of the nursing staff and increase their working enthusiasm. It also contributes to relieving the doctor-patient relationship. It was indicated in the results of this research that the quality-of-life scores for various items of the patients in observation group were obviously higher than those in control group, which is inseparable from the roles of comprehensive nursing intervention in eliminating the patients’ unhealthy emotions during hospitalization, keeping optimistic thinking, improving the self-confidence of the patients and making the patients and their families cooperate with the treatment positively and actively. The statistical report of Chan DKC et al (19) showed that keeping an optimistic and confident thinking may be greatly helpful to the treatment of a variety of diseases. Furthermore, it was revealed in a study (20) that comprehensive nursing intervention can enhance health education to the patients, reduce their feeling of uncertainty about the disease and increase their information and motivation about cure of disease. The comprehensive nursing intervention requests the nursing staff to pay close attention to various test indexes and clinical symptoms of the patients and report to the physician in charge immediately when there are abnormal physiological indexes or deterioration of disease in the patients, so as to perform corresponding treatment (21, 22). The occurrence of complications is avoided effectively, and the prognosis of the patients is improved by means of real-time information feedback and timely treatment. It was also revealed in this research results that the comprehensive nursing intervention could notably lower the incidence of postoperative complications in the patients, which plays a positive role in improving the treatment effect of patients. Additionally, the analysis results of the 20-month survival rate showed that the rate of the patients receiving comprehensive nursing intervention was elevated remarkably.

In this research, only the short-term survival and prognosis of the patients were assessed due to the limit of time. Meanwhile, the sample size was small, and the sample source was narrow in this retrospective analysis, so the results were not representative. With regard to diversified types of interventional operations at present, further studies on whether the nursing mode adjusted according to the surgical approach is more conducive to improving the treatment effect need to be performed.

## Conclusion

Comprehensive nursing intervention can notably improve the life quality of the patients after the interventional therapy for liver cirrhosis and liver cancer, lower the incidence of complications after the interventional therapy and increase the survival rate of the patients, which is worthy of clinical popularization and application.

## Ethical considerations

Ethical issues (Including plagiarism, informed consent, misconduct, data fabrication and/or falsification, double publication and/or submission, redundancy, etc.) have been completely observed by the authors.
